# Acupuncture and Traditional Chinese Veterinary Medicine in Zoological and Exotic Animal Medicine: A Review and Introduction of Methods

**DOI:** 10.3390/vetsci9020074

**Published:** 2022-02-09

**Authors:** Tara M. Harrison, Sarah M. Churgin

**Affiliations:** 1 College of Veterinary Medicine, North Carolina State University, Raleigh, NC 27607, USA; 2 Ocean Park, Hong Kong, China; vetadventurer@gmail.com

**Keywords:** acupuncture, zoo animal, exotic animal, non-domestic animal, electroacupuncture, aquapuncture

## Abstract

Acupuncture has been used extensively in domestic animal medicine to treat a variety of medical conditions and diseases as an adjunct or primary therapy. Exotic animals are becoming increasingly common as pets. Owners are expecting therapies for these non-domestic animals to be similar to those available to their other domestic pets. Additionally, zoological and aquatic facilities provide medical care for the entire lives of the animals that are housed in their facilities. Many conditions similar to those observed in domestic animals can develop in zoological species and can benefit from treatment with acupuncture. Through operant conditioning or routine medical examinations, the use of acupuncture as an adjunct therapy is becoming more common. The following paper presents a summary of the types of non-domestic animals treated, for which conditions, and how these methods are commonly used.

## 1. Introduction

Acupuncture in animals initially started with Zhao Fu and Bo Le [1,2]. Zhao Fu initially started using hemo-acupuncture at Jing-mai to treat diseases in horses during the Zhou-mu-gong period (947 to 928 B.C.) [2]. Bo Le, who lived during the Qing-mu-gong period (659–621 B.C.), treated horses, specifically racehorses, and was considered to be an equine specialist at the time [2]. Perhaps one of the first non-domestic animals to be treated with Traditional Chinese Veterinary Medicine (TCVM) was the elephant [3]. Additionally, TCVM has been used in laboratory animals over the years as a part of peer-reviewed literature and as part of evaluating its use in humans and domestic animals [4,5,6,7,8,9,10,11,12,13,14].

The use of acupuncture, or TCVM, has gained popularity as a choice of therapy due to widespread opiate shortages, the desire to minimize the use of opiates in veterinary medicine, and in the pursuit of additional multimodal tools to treat patients [15]. Acupuncture has grown to be an accepted method of treatment for domestic animals, such as horses, dogs, and cats, and has been used as an adjunctive therapy in numerous other animals [2,16,17,18,19,20,21,22,23,24,25,26,27,28,29,30,31,32,33,34,35,36,37,38,39]. Unfortunately, there are limited studies on meridians and their use in large varieties of species. However, non-domestic animals, or exotic animals, are becoming increasingly popular pets and the authors have noticed that their owners are seeking veterinary care of a similar caliber to that of domestic animals.

Zoological species under human care at institutions, such as zoos and aquaria, are also benefiting from extended lifespans, which is attributed to improved husbandry, nutrition, and medical care [40]. As zoological medicine has advanced, veterinarians are seeking additional multimodal modes of therapy in treating these animals’ medical conditions. Acupuncture may have been primarily used in zoological animals in the past during anesthetic procedures. However, with advances in the field of behavioral training, acupuncture is now possible in many animals without anesthesia through operant conditioning, which is a method of training animals through positive reinforcement to accept medical procedures, such as injections or needle placement.

## 2. Materials and Methods

A complete literature review was conducted using PubMed, Web of Science Zoological Record (via Web of Science) from the dates of database inception (1983 for PubMed, 1973 for CAB, and 1865 for Zoological Record). PubMed, Web of Science Zoological Record were searched using the following root words of acupuncture and zoo: avian, reptile, hoofstock, rabbit, guinea pig, ferret, amphibian, lizard, turtle, tortoise, non-domestic felid, non-domestic canid, tiger, elephant, and wildlife. The entire collection of American Journal of Traditional Chinese Veterinary Medicine publications was manually searched for any non-domestic animal references. All manuscripts that fit the search criteria of acupuncture, and at least one non-domestic animal reference, were included in this review. Additional examples were provided from cases or common conditions that the authors have been involved in to supplement literature findings. Cases that involved experimental induction of a medical condition and the use of TCVM were excluded from use.

## 3. Results

In both the formal review of the literature, and in the authors’ experience, it is evident that acupuncture in exotic and zoological species is increasing in use and within publications over time. However, there are still minimal publications and books based on TCVM and acupuncture in non-domestic animals.

Despite the lack of published literature existing for numerous non-domestic species, the authors have used acupuncture as an adjunctive or primary therapy in many of these species with no adverse effects and have observed positive clinical responses. Despite meridians not being entirely known or mapped for all species, they can be approximated and used successfully in a number of species through transposition from domestic animals [41]. The species with cases published in the literature include elephants [42], rabbits [43], rodents [43], avians [33,43], tigers [36,44], snakes [45], and turtles [34,35], among others. Approximate meridians have been printed in elephants [46], birds [37,47], rabbits [26], dolphins [48,49], and fish [50,51]. Approximate acupoints have been identified in a boa constrictors as well [52]. Transposition of points has been carried out in a variety of species, including rabbits, ducks, chickens, and dolphins [41,49]. The following are summaries of the available literature, the methods of treating a variety of non-domestic animals, and examples of conditions for which acupuncture is commonly used in these species.

### 3.1. Amphibians

Acupuncture is not commonly used in amphibians, although it is possible to use it for certain situations. There is no published literature on the use of acupuncture in amphibians, although a study evaluating the use of photobiomodulation for wound healing exists [53]. Acupuncture has been used by the authors utilizing a method of approximate transposition of canine points to amphibian anatomy. The typical TCVM methods of diagnosis, such as evaluating the tongue color and pulse characteristics, may be stressful and inaccurate in these species. The medical conditions that can be treated in amphibians are typically osteoarthritis, paresis, or paralysis (Table 1).

**Figure 1:**
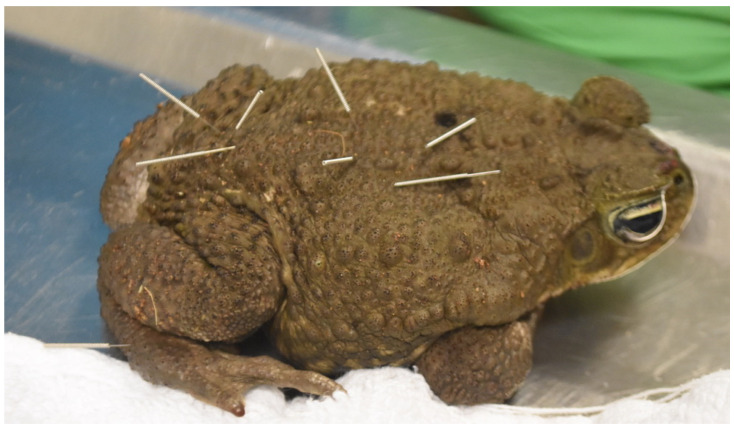
Photo of a toad receiving acupuncture with 42-gauge hand needles.

A special consideration for performing acupuncture in amphibians is to minimize potential damage to the skin, which can be very thin and susceptible to infection. Amphibian skin can occasionally contain toxins that can affect humans, therefore, powder-free gloves should be worn while handling amphibians [54]. It is also best to rinse the gloves in a portion of the animal’s tank water prior to handling the animal in order to minimize the risk of skin trauma. In general, medial and ventral acupuncture points are challenging to access in these species due to their close proximity to the ground in their natural positioning and holding the animal upright, or in dorsal recumbency, in order to access medial and ventral points can induce stress. For predominantly aquatic species, it may be more of a challenge to treat and to keep the needles in the animal; additionally, electroacupuncture should not be used in these species. The needles that tend to work best for acupuncture are the 42-gauge hand needles for a 3 to 5 min long session (Figure 1).

### 3.2. Reptiles

**Figure 2:**
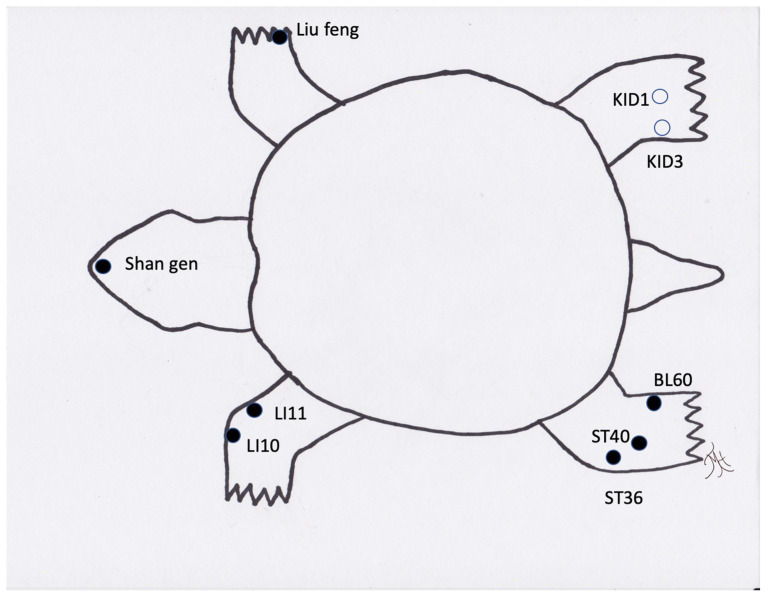
Generic image of a turtle/tortoise and common acupuncture points. Note those in an open circle are located on the ventral/medial portion of the limb.

**Figure 3:**
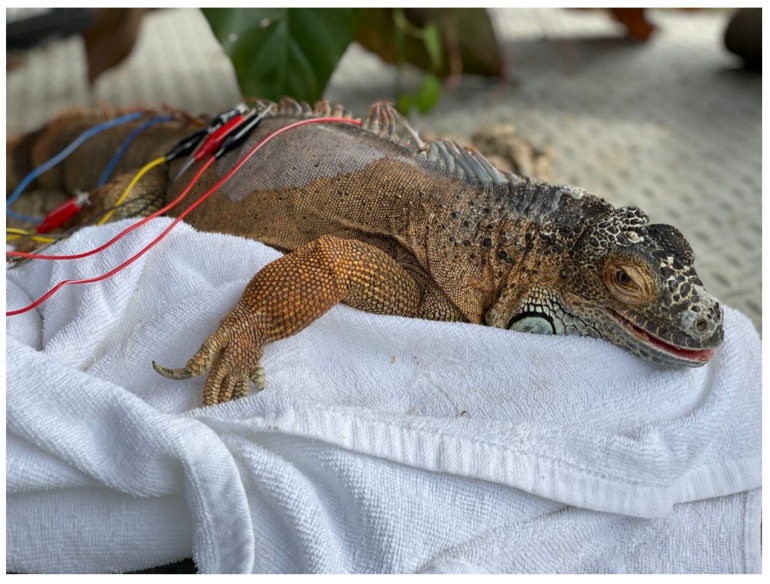
Photo of an iguana receiving acupuncture and electroacupuncture.

**Figure 4:**
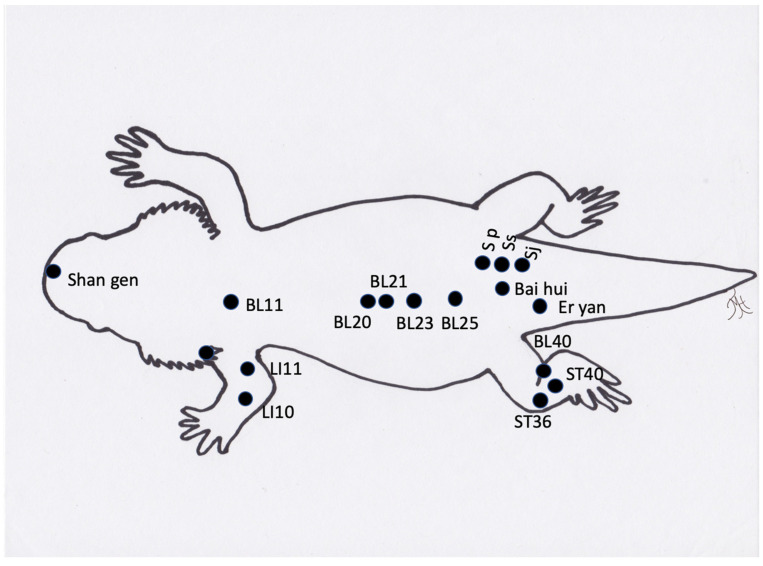
Generic image of a bearded dragon lizard and approximate common acupuncture point locations.

Reptiles present unique considerations for acupuncture as certain acupuncture points are not accessible due to the anatomy of certain species (i.e., points along the spine in turtles (bladder meridian) (Figure 2) or those on the limbs in the case of snakes). Additionally, the methods typically used to make a TCVM diagnosis, such as through the use of tongue color and pulse, appear to be generally inaccurate, or not possible, in reptiles. The determination of acupoints has made some progress through an initial study to approximate acupoints in a boa constrictor using an electrostimulator [52]. Although these points were not identified with a meridian, they are still areas that likely pertain to acupoints. Overall, reptiles appear to respond well to acupuncture therapy (Figure 2, Figure 3 and Figure 4). Reptiles also respond extremely well to electroacupuncture and often become noticeably more relaxed once the stimulation is added, even if they initially resent the insertion of needles.

Acupuncture use in reptiles has predominantly been used for anesthesia recovery [34,35], painful conditions [55], and stimulation of appetite.

**Figure 5:**
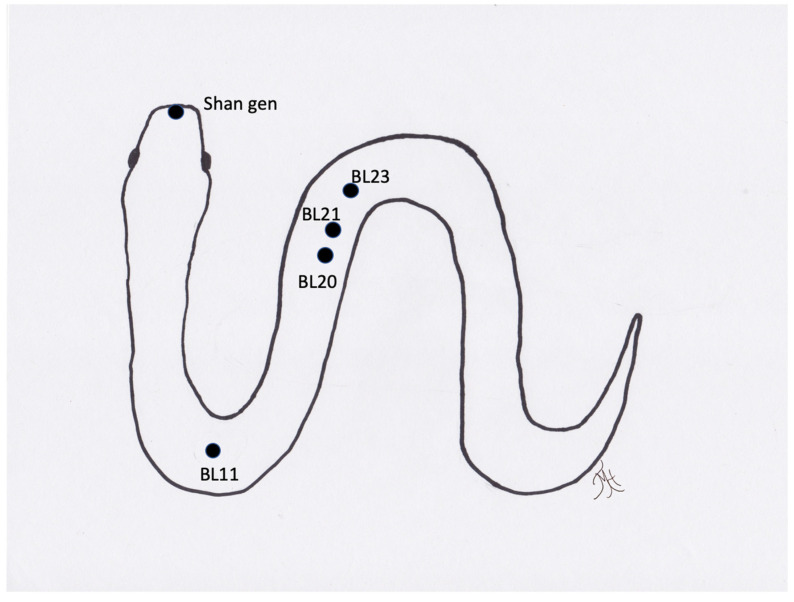
Generic image of a snake and approximate location of acupuncture points.

When using acupuncture to recover reptiles from anesthesia, it was found that acupuncture significantly decreased the recovery times compared to the negative controls [34,35]. Acupuncture has also been reported to treat locomotive concerns in a tortoise [38], and Komodo dragon [55]. A boa constrictor has also been treated with an integrative approach using acupuncture for treatment of a recurrent respiratory infection [45], and for wound healing, including the use of moxibustion [56]. Lizards have benefitted from acupuncture and electroacupuncture when suffering from paresis, paralysis, nutritional secondary hyperparathyroidism, scoliosis, and kyphosis (TMH experience). Another condition that is successfully treated is inappetence. The exact acupuncture points may be challenging to determine in species such as snakes (Figure 5), but acupuncture based on region and approximating of transposing points or inserting needles (cranial and caudal) into lesions, such as in spondylosis or spondylitis, appears to be adequate in these species. One author (SMC) has treated two long-term reptile cases in a zoological institution with acupuncture, including a geriatric green iguana with inappetence and poor activity level and a king rat snake with severe spinal osteopathy resulting in poor mobility. Both of the animals were treated weekly for many months with both dry needles and electroacupuncture, and both responded positively. In the case of the iguana, the treatment sessions were coordinated with the research department at the institution and the research team developed a behavioral ethogram in order to monitor and demonstrate the effects of the acupuncture (publication in preparation). Acupuncture in reptiles is best performed by inserting the needles between the scales, so that the scales are not damaged and to prevent future dysecdysis. In smaller species, 42-gauge hand needles are easiest to use. In larger species, larger gauge needles can be used. The length of time for treatment depends on the animals, but generally 3 to 5 min or longer with electroacupuncture can be achieved.

### 3.3. Avians

Acupuncture is not as commonly used in avian species as in other species, however, when used, most birds respond well to acupuncture [33,37,57]. There are a vast number of avian taxa and a great diversity between them. In general, in attempting to make a TCVM diagnosis, observations of the tongue and pulse can be performed, but it may be extremely stressful for the patient and may be inaccurate as many species have pigmented tongues. Different from many nontraditional species, an avian acupuncture meridian charts exists [47]. A variety of methods are used in the treatment of birds, including inserting needles and leaving them in, or just putting needles in, manipulating them, and removing the needles immediately [47]. In some species, it is substantially easier to use aquapuncture. The anatomical differences between bird species can make accessing certain points challenging or impossible. For instance, while some species, such as cranes and chickens, have long legs with easily-accessible and identifiable anatomy, other species, such as parrots and penguins, have short legs with much of the femur tucked under the body in an inaccessible area. Additionally, when performing aquapuncture in avians, the clinician should be aware of the air sac locations, so as to not induce a fatal aquapuncture injection directly into an air sac, which is most commonly carried out when attempting subcutaneous injections along the back of the bird, and should be avoided. Some gallinaceous birds, such as chickens and peafowl, tolerate needles quite well and have been observed by the author (TMH) to exhibit a deqi response, such as yawning, with a response to therapy within one to two sessions.

When using needles for acupuncture on smaller birds in particular, it is easiest to use colored handled needles so that it is easier to see them. Non-colored needles can be easily confused with feather shafts and, therefore, make it challenging to re-locate the needles. Commonly used needle sizes are 32- or 34-gauge needles in medium to larger birds and 36-gauge needles in smaller birds. The length of the treatment session is typically 3 to 5 min or less, unless the bird is habituated to treatment, in which case longer treatments could be accomplished.

**Figure 6:**
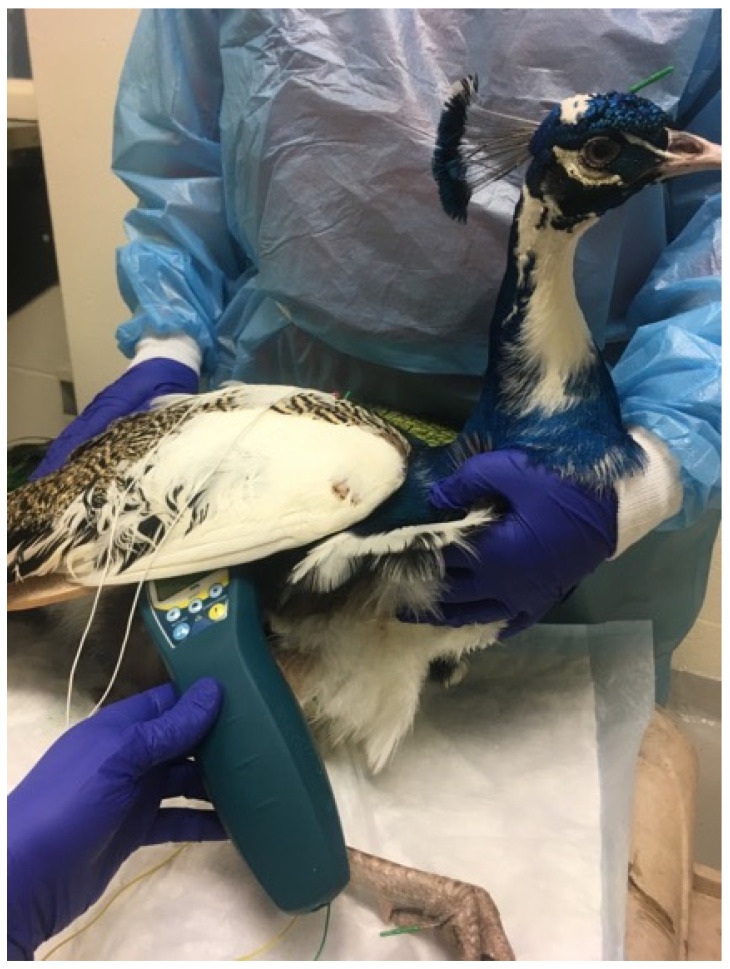
Photo of a peafowl receiving acupuncture, electroacupuncture, and photobiomodulation for treatment of appetite stimulation and paresis of the limbs.

**Figure 7:**
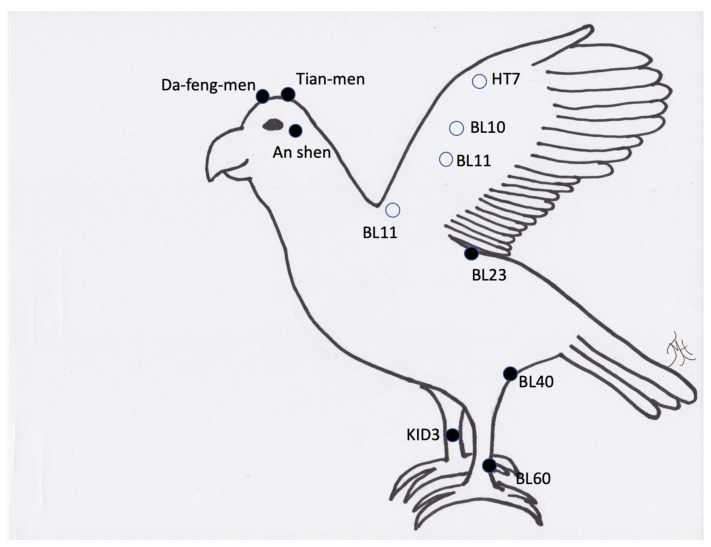
Generic image of a bird and common acupuncture point locations. Note the open circles are located on the other side of the wing (lateral surface) or in the case of BL11 just lateral to the spine on the other side of the wing.

Conditions commonly treated in birds include osteoarthritis, paresis, anorexia, egg binding, and pododermatitis (Figure 6 and Figure 7). Feather-destructive behavior, or feather plucking, can be treated in conjunction with other treatment and diagnostic modalities, such as behavioral modification, habitat changes, and enrichment [47]. The use of the acupuncture point An Shen, located along the midpoint of the ear and directly caudal to the ear opening in avians, is a great aquapuncture location to calm birds for hours or even days. In order to use this point, one author (TMH) uses a subcutaneous 50:50 mixture of saline and Vitamin B12 proportionate to the size of the bird, i.e., for small birds 0.05 mL SQ in this location and up to 0.1 mL in larger birds. One author (SMC) has also used tian-men or da-feng-men in several avian species as a calming point prior to insertion of other needles and found that it consistently produces a calming effect.

### 3.4. Small Mammals

Acupuncture in small mammals, such as rabbits, has been performed for a number of years as a laboratory model for acupuncture in humans [14,58,59,60,61,62,63,64]. These animals generally tend to accept acupuncture, electroacupuncture, and aquapuncture [10,12,43,65,66,67,68]. Because these animals are prey species and often have small oral cavities, evaluating the tongue can be very stressful and inaccurate. Similarly, it may be challenging to assess pulse, but of all of the methods of TCVM diagnostics, this is the most feasible method. Most of these animals respond well to bladder meridian or proximal limb points in the authors experience. They tend to be less tolerant of lower limb points and medial points are very challenging to access or stressful to use. The conditions treated typically include spinal injuries, osteoarthritis, musculoskeletal injuries, and gastrointestinal disorders [69].

**Figure 8:**
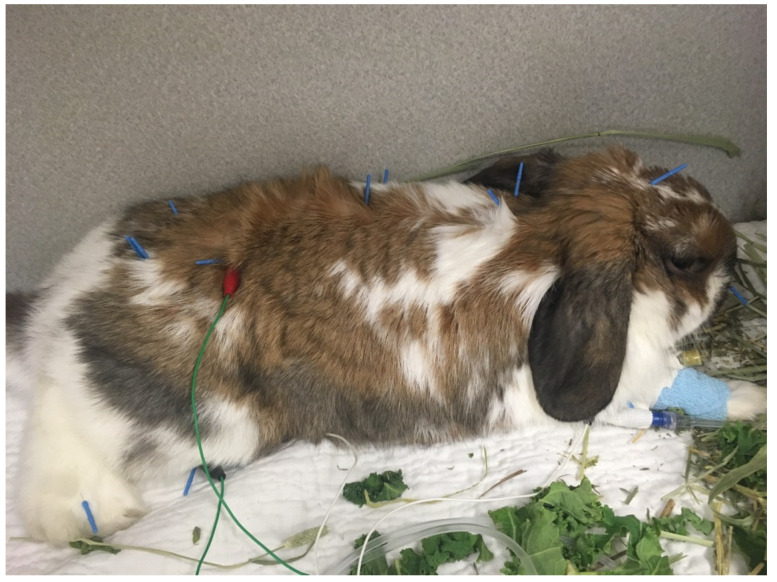
Photo of a rabbit receiving acupuncture with 36-gauge needles for gastrointestinal stasis.

**Figure 9:**
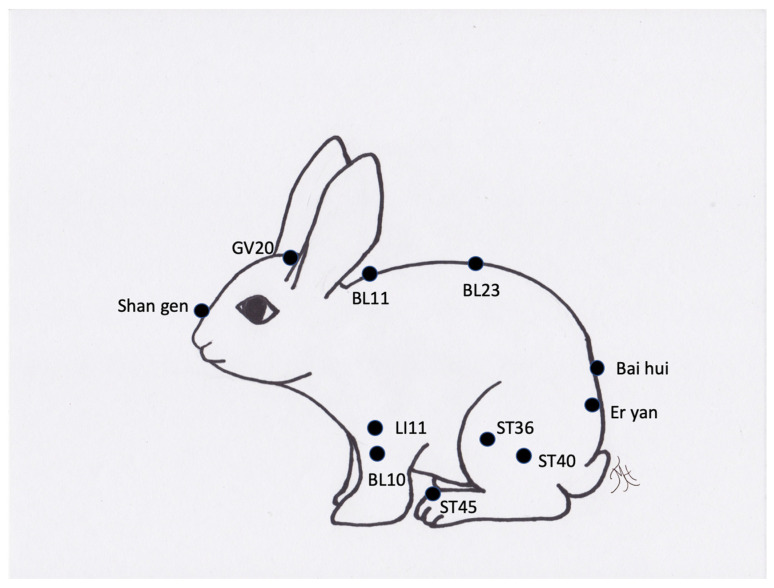
Generic image of a rabbit and approximate common acupuncture point locations.

Perhaps the most commonly treated conditions treated are osteoarthritis or spinal lesions in rabbits, or gastrointestinal stasis in rabbits and guinea pigs [14]. Similar to equines, stasis patients greatly benefit from acupuncture, electroacupuncture, and aquapuncture and tend to respond well to treatment unless the gastrointestinal stasis is caused by a more severe situation, such as a mechanical obstruction (Figure 8 and Figure 9).

Ferrets, on the other hand, are very challenging to dry needle and, overall, are minimally tolerant of acupuncture and electroacupuncture. Although electroacupuncture has been used experimentally to evaluate the treatment of emesis in ferrets successfully [70,71], clinically, it can be challenging to use in the authors’ experience. Aquapuncture is typically more successful in ferrets and can be used to treat various conditions, such as nausea, pain secondary to trauma, or as a symptomatic treatment in neoplasia cases. Osteoarthritis and spinal conditions tend to be uncommon in ferrets. Ferrets greatly benefit from a “less is more” approach to treatment, and it is advisable to start with a small number of points and to work up in numbers of acupuncture points over time.

When needling small mammals, colored needles tend to be easiest to find as their fur can be very fine and, therefore, hide copper or steel needles. The most commonly used needles tend to be 36- or 34-gauge for a therapy period of 5 to 10 min, except in ferrets, which generally benefit from a substantially shorter treatment time.

### 3.5. Zoological Animals

**Figure 10:**
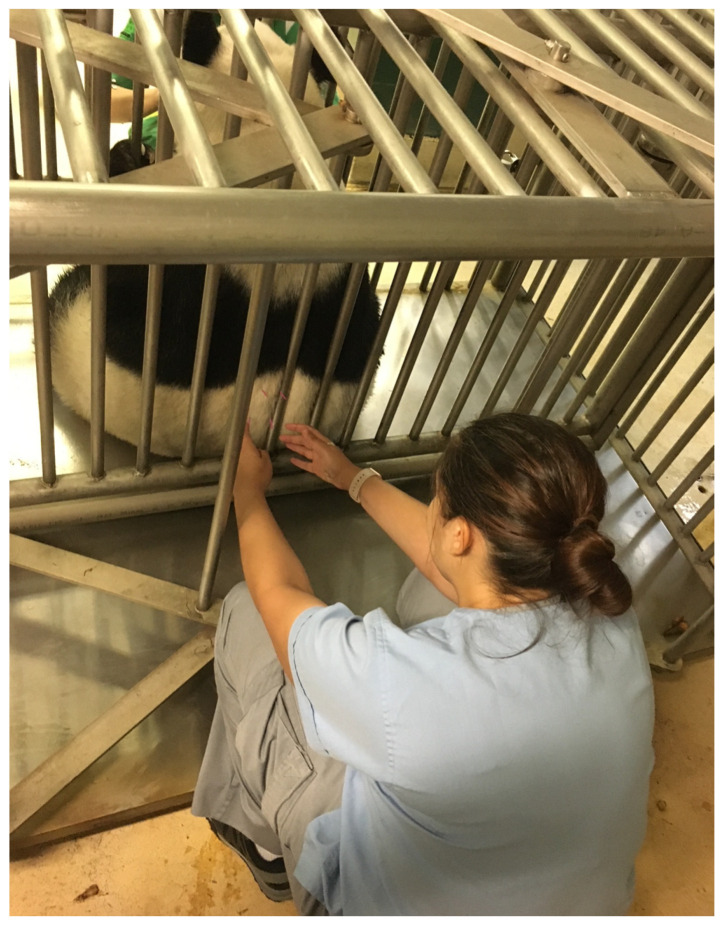
Photo of a giant panda receiving acupuncture through operant conditioning while in a squeeze chute.

There are many species of zoological animals that may benefit from acupuncture therapy. Zoological animals under managed care have benefited in recent years from improved husbandry, nutrition, and medical care. As a result, these animals tend to live long lives and develop geriatric medical conditions, such as osteoarthritis, cancer, and gastrointestinal concerns [40]. However, many animals in zoos and aquaria are involved in some sort of operant conditioning or training program and it is often possible to train animals to accept injections. The use of acupuncture needles or aquapuncture is an easy transition for those animals trained to accept injections. Even in the most well-trained animal, however, treatment with acupuncture typically involves gradually increasing the number of points and the duration of the treatment sessions over time. The benefits of acupuncture and additional numbers of needles must be weighed against the risks of losing a needle if the animal unexpectedly leaves during the treatment session. Often, especially in the case of dangerous animals, specialized equipment is used to aid in keeping the acupuncturist, the handlers, and the animal safe and secure, such as squeeze chutes (Figure 10). In animals that are not trained to accept needles, or those that cannot be restrained to facilitate needle insertion, acupuncture may be performed under anesthesia. When treating an animal under anesthesia, more points, therapies, and a longer length of the session are possible. When treating an animal under anesthesia, it is best to avoid anesthetic reversal with μ- and δ-opioid receptor antagonists, as some, but not all, of the effects of acupuncture may be reversed [72]. Of course, the risks of anesthesia must be weighed against the possible benefits of acupuncture. In the authors’ experience, an animal might be anesthetized for acupuncture if there are other important procedures to perform for that animal, but it would be uncommon to anesthetize a zoo animal for the purpose of acupuncture alone.

**Figure 11:**
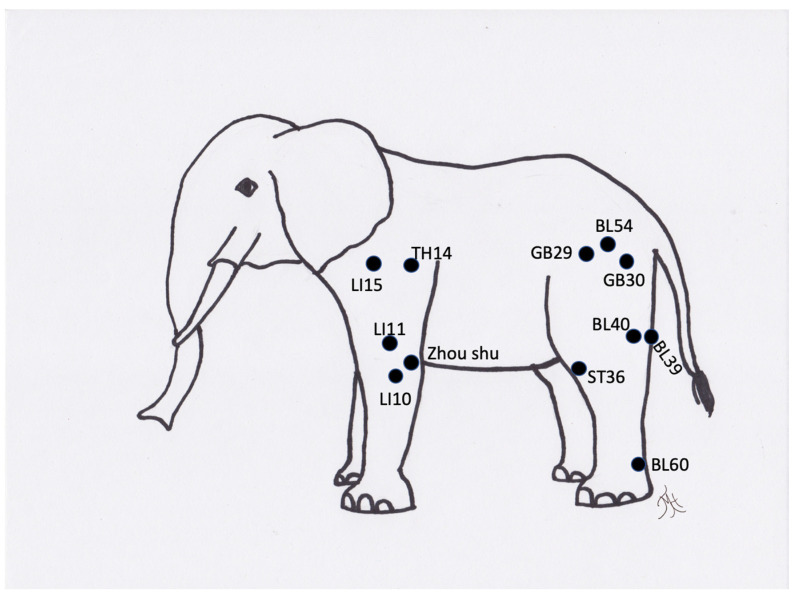
Generic image of an elephant and approximate common acupuncture point locations.

Perhaps the most commonly treated zoological-type animals are elephants [3,42]. The meridian points have been mapped out for elephants and they have been treated for painful or other arthritic lesions for many years (Figure 11) [46]. Treating elephants can be challenging in many ways, particularly from a safety aspect. Most elephants currently under human care are managed through protected contact, meaning that humans are always separated from the animals by a barrier, and the elephants are trained to come up to the edge of the holding facility. In this method of treatment, the acupuncturist would need to reach through the barriers to treat the animal. This requires positioning of the animal in a method that facilitates close contact to place needles, as well as training the animal to accept the acupuncture. The medial points are not safely accessible in most of these animals. The bladder meridian points may also be hard to access due to the height of the animal, although stepping stools or other tools could aid in this treatment. Needles, such as 26-Ga colored handled acupuncture needles, are easy to use and should be inserted through the cracks in the skin. Elephants can remove needles, therefore, having a needle with a colored handle assists in locating the needle after self-removal. The treatment time can vary depending on the training of the elephant, but typically starts at 5 min in length and could last for 15 min or more.

Carnivores also benefit from acupuncture. The methods of therapy will depend on the size and the type of carnivore. Small canids or felids may be able to be manually restrained for acupuncture or photobiomodulation therapy, but depending on the animal, this could be a very stressful situation. In the smaller carnivorous animals, it may not be possible to check the tongue color safely, but it may be possible to palpate a pulse for a TCVM diagnosis. Larger carnivores, such as lions or bears, need to be treated through training methods of stationing next to a barrier or must be treated under general anesthesia and evaluation of tongue color may be performed, however, pulse is typically not safely obtained for a TCVM diagnosis. Certain species, such as black bears, are prone to osteoarthritis [73]. One author (TMH) has treated a black bear long-term for osteoarthritis with acupuncture, aquapuncture, and photobiomodulation. This animal was treated in a squeeze chute initially with aquapuncture to train it to accept acupuncture. This animal was treated every 2–4 weeks for several years. Many larger felid species have also been commonly reported to have spinal conditions and osteoarthritis and have been treated for these conditions [36,44]. Carnivores in particular can be very dangerous to conduct acupuncture on, and depending on the animal, the acupuncturist may only be able to treat one point at a time and then gradually increase the number of points. In general, the medial points are not accessible due to safety reasons and cannot be accessed in carnivores unless they are anesthetized or can be easily manually restrained. The treatment sessions tend to be short in length unless the animal is anesthetized.

One author (SMC) has performed long-term, ongoing acupuncture using dry needling in both a giant panda and a red panda. Both of the animals have been treated for geriatric concerns, such as osteoarthritis and reduced mobility. The giant panda was treated using a specialized squeeze cage that allowed the animal to extend its arm onto a small platform outside of the cage, which was originally designed to allow for venipuncture. Over time, the animal was trained to accept acupuncture needles in the elbow and forearm points. Later, spinal points were added but the animal had to be in a different position utilizing the squeeze function of the squeeze cage to allow for the needle placement. The red panda, on the other hand, was trained to lie in sternal recumbency and allow the treatment of both spinal points and hip points, for 4–5 min per session. No restraint was needed for the red panda. The positive effects were noted in both of these animals.

Hoofstock also can benefit from acupuncture [32]. The evaluation of the tongue and pulse can be challenging or dangerous to access for a TCVM diagnosis. Some hoofstock, such as camelids, have been reported to have benefitted from acupuncture in the literature [74]. For treatment considerations, many larger hoofstock, such as giraffe, are routinely trained to accept injections and to position themselves next to barriers for treatment. The ability to treat other hoofstock, such as cervid, bovid, or equid species, may be entirely dependent on the temperament of the individual. Many hoofstock, however, may only be treatable while under general anesthesia. The medial points are not accessible unless the animal is anesthetized. Other megavertebrates, such as rhinoceros, may also be trained to accept injections and are treatable with acupuncture. Due to the extreme thickness of their skin in certain areas, treating the lateral limbs and the neck may be more feasible than along their spine. The medial points are accessible in some animals, but not safely in others depending on the animal. The evaluation of the tongue and pulse are generally not safe to access for the rhinoceros. The conditions that are commonly treated in hoofstock and megavertebrates are osteoarthritis, other painful conditions, and gastrointestinal issues, such as colic.

Other animals, such as anteaters and koalas, have been treated by the authors (and personal communication, P. Brock 2021) for osteoarthritis and bone injuries. These animals can be treated in limited settings while they are either perched on a branch as in the koala, or while in their enclosure area (anteater). For both of these species, they do accept aquapuncture and electroacupuncture, as well as photobiomodulation therapies, while they are awake. Primates have also been reported to have been treated with acupuncture in laboratory animal medicine [4], but through operant conditioning this could be attempted in a clinical zoological settings as well. The biggest challenge in treating primates with acupuncture through training is their natural dexterity and ability to self-remove the needles.

Other non-domestic animals, such as a dolphin, have been treated with TCVM [48], although predominantly they have been treated with herbal medicine [75]. Acupuncture points have been mapped for dolphins but are not utilized to the authors’ knowledge [48,49]. The use of tongue color could be accessible for trained animals, but obviously a femoral pulse is not possible due to their anatomy for a TCVM diagnosis. Both cetaceans and pinnipeds are exquisitely amenable to training and could certainly be trained to accept acupuncture, but the greatest risk is in the animal leaving the training session with the needles still in place and then losing needles in an aquatic environment. This could potentially pose a risk to other animals in the pool. Thus, treatment may be encouraged to be performed with the animal “hauled out” or voluntarily stationed on a land area in the case of cetaceans, or temporarily “dry-docked” and separated from the pool in the case of pinnipeds. The acupuncturist would need to remain in a position near to the trainer and would not be able to move around extensively, which would only allow them to have to access to the points near where they are positioned. The duration of the treatment would likely vary by animal and would be gradually increased over time. Aquapuncture is likely a good option for marine mammals to avoid the risks associated with keeping a needle in place in the aquatic environment.

## 4. Discussion

As presented, acupuncture can and has been used in a variety of non-domestic animals. Probably the most recognized use of acupuncture, emergency resuscitation [76], has been used in zoological species, but as demonstrated in the cases and conditions above, its use is not limited to that. Acupuncture has become a part of a multimodal approach to analgesia and, as a study on myofascial muscle pain in rabbits that demonstrated improved pain relief through affecting endogenous opioids has shown, additional use of acupuncture as a multimodal approach is warranted [65].

Often, the only thing keeping veterinarians from using acupuncture on non-domestic animals is the lack of a veterinary acupuncturist qualified to treat them. Qualifications are generally not substantially more than a typical equine or canine acupuncture certification. The individuals involved in treating these animals need to understand the general anatomy, physiology, and limitations for treating the animal. Much of this information can be garnered through a review of anatomy and medical conditions in books, meeting with husbandry staff, asking targeted questions, and viewing the animal’s living area and any restraint devices. Creativity is almost always required. The importance of involving training staff in designing the treatment plan cannot be overstated, and in fact, empowering the training staff to help with treatments will also often result in more effective treatments. It is important to minimize stress throughout the treatment and, as such, obtaining a full TCVM diagnosis may not be possible prior to treating the animal. Furthermore, the treatments may need to be abbreviated compared to typical domestic animal treatments in order to ensure that the treatment session is a positive experience and that the animal will be willing to be treated again. A great deal of patience is required for both the acupuncturist and the training staff, and it is important to educate the staff on expectations, such as slower or less obvious benefits, initially. When possible, the use of objective monitoring sheets with scoring systems may be useful in monitoring the subtle changes over time.

Acupuncture meridians exist for some nontraditional animals but additional research and evaluation of anatomy of these animals would greatly assist in attempting to develop additional meridian charts for these species. Additional peer-reviewed research on the use of acupuncture in a variety of these cases would also be beneficial. Although case reports do exist for several non-domestic animals, case-series publications or case-controlled research would increase the information available for clinicians to use for future treatment of similar cases.

## 5. Conclusions

In conclusion, acupuncture can be given to numerous non-domestic animal species to greatly benefit their welfare and conditions. Through training methods, such as operant conditioning and a lot of patience and creativity, many TCVM methods can be utilized to improve the health and wellbeing of these animals and should be utilized as part of multimodal forms of therapy.

## Figures and Tables

**Table 1:** Table of examples of a variety of zoological and exotic animals and common conditions that may be treated with acupuncture, with examples of what types of points could be available and usable for each condition.

Species	Common Conditions Acupuncture Is Used For	Potential Main Points
Amphibian	Arthritis	BL11, BL23, BL54, GB29, GB30, BL40, BL60, KID3, Bai Hui, Er Yan, Shen-Shu, Shen-peng, Shen-jiao
Amphibian	Paresis/Paralysis	KID1, PC8, BL11, BL23, Liu-feng
Snake	Spondylosis/Spondylitis	BL meridian cranial and caudal to region
Snake	Anorexia	BL meridian approximate ½ length to approximate ST and SP points, Shan-gen
Lizard	Arthritis	BL11, BL23, BL54, GB29, GB30, BL40, BL60, KID3, Bai Hui, Er Yan, Shen-Shu, Shen-peng, Shen-jiao
Lizard	Anorexia	Shan-gen, LI10, LI11, ST36, ST40, BL20, BL21, BL25
Turtle/Tortoise	Anorexia	Shan-gen, LI10, LI11, ST36, ST40
Turtle/Tortoise	Paresis hind limb	BL40, BL60, KID3, KID1, Liu-feng
Avian	Wing arthritis	LI10, LI11, HT3 (+/−), SI4, BL11(advanced avian skill), BL 23 (advanced avian skill)
Avian	Limb arthritis	BL11(advanced avian skill), BL 23 (advanced avian skill), BL40, BL60, KID3
Avian	Appetite stimulation	ST36, ST40, ST45, Shan-gen
Avian	Feather plucking	An Shen, HT7, LI11, ST36, and points for pain near picking
Avian	Pododermatitis	LIV8, GB34, circle the dragon
Rabbit/Guinea Pig	Gastrointestinal stasis	LI10, LI11, ST36, ST40, ST45, Shan-gen
Rabbit/Guinea Pig	Spinal arthritis	BL11, BL23, Bai-hui, Er-yan, cranial and caudal to lesion
Rabbit/Guinea Pig	Pelvic/hind limb arthritis	BL11, BL23, Bai-hui, Er-yan, Shen-shu, Shen-peng, Shen-jiao, BL54, GB29, GB30, BL40, BL60, KID3
Megavertebrate	Arthritis front limb	LI10, LI11, LI15, TH14, Zhou-shu
Megavertebrate	Arthritis hind limb	ST36, BL54, GB29, GB30, BL39, BL40, BL60
Carnivore	Arthritis front limb	LI10, LI11, LI15, TH14, Zhou-shu
Carnivore	Arthritis hind limb	ST36, BL54, GB29, GB30, BL39, BL40, BL60, KID3
Carnivore	Spinal arthritis	Likely under anesthesia, points cranial and caudal to lesion, BL11, BL23, Bai-hui, Er-yan, BL39, BL40, KID10, BL60, KID3
Hoofstock	Arthritis front limb	LI10, LI11, LI15, TH14, Zhou-shu
Hoofstock	Arthritis hind limb	ST36, BL54, GB29, GB30, BL39, BL40, BL60
Hoofstock	Gastrointestinal	Shan-gen, LI10, LI11, ST36, ST40, BL20, BL21, BL25 (depends on height of animal as to success of obtaining all BL points)

## Data Availability

The data presented in this study are available in the manuscript.
